# Progress and application of lung-on-a-chip for lung cancer

**DOI:** 10.3389/fbioe.2024.1378299

**Published:** 2024-05-24

**Authors:** Lantao Li, Wentao Bo, Guangyan Wang, Xin Juan, Haiyi Xue, Hongwei Zhang

**Affiliations:** ^1^ Department of Anesthesiology, Sichuan Clinical Research Center for Cancer, Sichuan Cancer Hospital and Institute, Sichuan Cancer Center, Affiliated Cancer Hospital of University of Electronic Science and Technology of China, Chengdu, China; ^2^ Department of Hepatopancreatobiliary Surgery, Sichuan Clinical Research Center for Cancer, Sichuan Cancer Hospital and Institute, Sichuan Cancer Center, Affiliated Cancer Hospital of University of Electronic Science and Technology of China, Chengdu, China; ^3^ Department of General Internal Medicine, Sichuan Clinical Research Center for Cancer, Sichuan Cancer Hospital and Institute, Affiliated Cancer Hospital of University of Electronic Science and Technology of China, Chengdu, China; ^4^ Department of Anesthesiology, West China Hospital, Sichuan University, Chengdu, China; ^5^ Department of Intensive Care Unit, Sichuan Clinical Research Center for Cancer, Sichuan Cancer Hospital and Institute, Affiliated Cancer Hospital of University of Electronic Science and Technology of China, Chengdu, China

**Keywords:** lung-on-a-chip, resistance mechanism, tumour microenvironment, nano drug delivery systems, ferroptosis

## Abstract

Lung cancer is a malignant tumour with the highest incidence and mortality worldwide. Clinically effective therapy strategies are underutilized owing to the lack of efficient models for evaluating drug response. One of the main reasons for failure of anticancer drug therapy is development of drug resistance. Anticancer drugs face severe challenges such as poor biodistribution, restricted solubility, inadequate absorption, and drug accumulation. In recent years, “organ-on-a-chip” platforms, which can directly regulate the microenvironment of biomechanics, biochemistry and pathophysiology, have been developed rapidly and have shown great potential in clinical drug research. Lung-on-a-chip (LOC) is a new 3D model of bionic lungs with physiological functions created by micromachining technology on microfluidic chips. This approach may be able to partially replace animal and 2D cell culture models. To overcome drug resistance, LOC realizes personalized prediction of drug response by simulating the lung-related microenvironment *in vitro*, significantly enhancing therapeutic effectiveness, bioavailability, and pharmacokinetics while minimizing side effects. In this review, we present an overview of recent advances in the preparation of LOC and contrast it with earlier *in vitro* models. Finally, we describe recent advances in LOC. The combination of this technology with nanomedicine will provide an accurate and reliable treatment for preclinical evaluation.

## 1 Introduction

Lung cancer, a global issue of widespread concern, has become a persistent public health challenge worldwide. More than 350 people die from lung cancer every day, which is the second leading cause of cancer death globally (Siegel et al., 2022). Based on population growth and ageing, the burden of cancer is expected to increase yearly. Current treatment modalities for lung cancer mainly include surgical resection, radiation, chemotherapy (Qin et al., 2018), and immunotherapy (Zhao and Cao, 2019). Chemotherapy, as an essential part of cancer treatment, is frequently constrained by drug solubility, poor distribution, low specificity, and a variety of side effects (Miller et al., 2019; Atmaca et al., 2022). Anti-tumour therapy becomes more challenging as tumour resistance rates climb. Anti-tumour drugs are frequently successful in the early stages of cancer, but after tumour recurrence, patients often develop variable degrees of resistance (Wang N. et al., 2023). Currently, the advent of targeted drugs can decrease the side effects of drugs, but they also face the problem of drug resistance, which may lead to tumour exacerbation (Zheng et al., 2019). In recent decades, the area of bio-nanoscience and cancer medicine has developed rapidly (Bjornmalm et al., 2017). Despite significant advances in some new therapeutic strategies, such as use of nanodrug delivery systems (NDDSs) (Briolay et al., 2021), clinical translation rates are low owing to the lack of efficient models *in vitro* for evaluating whole-body responses. The advent of microfluidic tumour-on-chip (TOC)-based systems offers a new approach to these challenges (Tian et al., 2022). Microfluidic devices offer a substantial reduction in reagent consumption, chemical reaction durations, and overall costs through integration on a single chip. This advancement holds the potential to facilitate innovative point-of-care (POC) and point-of-need solutions for clinical diagnostics (Gurkan et al., 2024).

**FIGURE 1:**
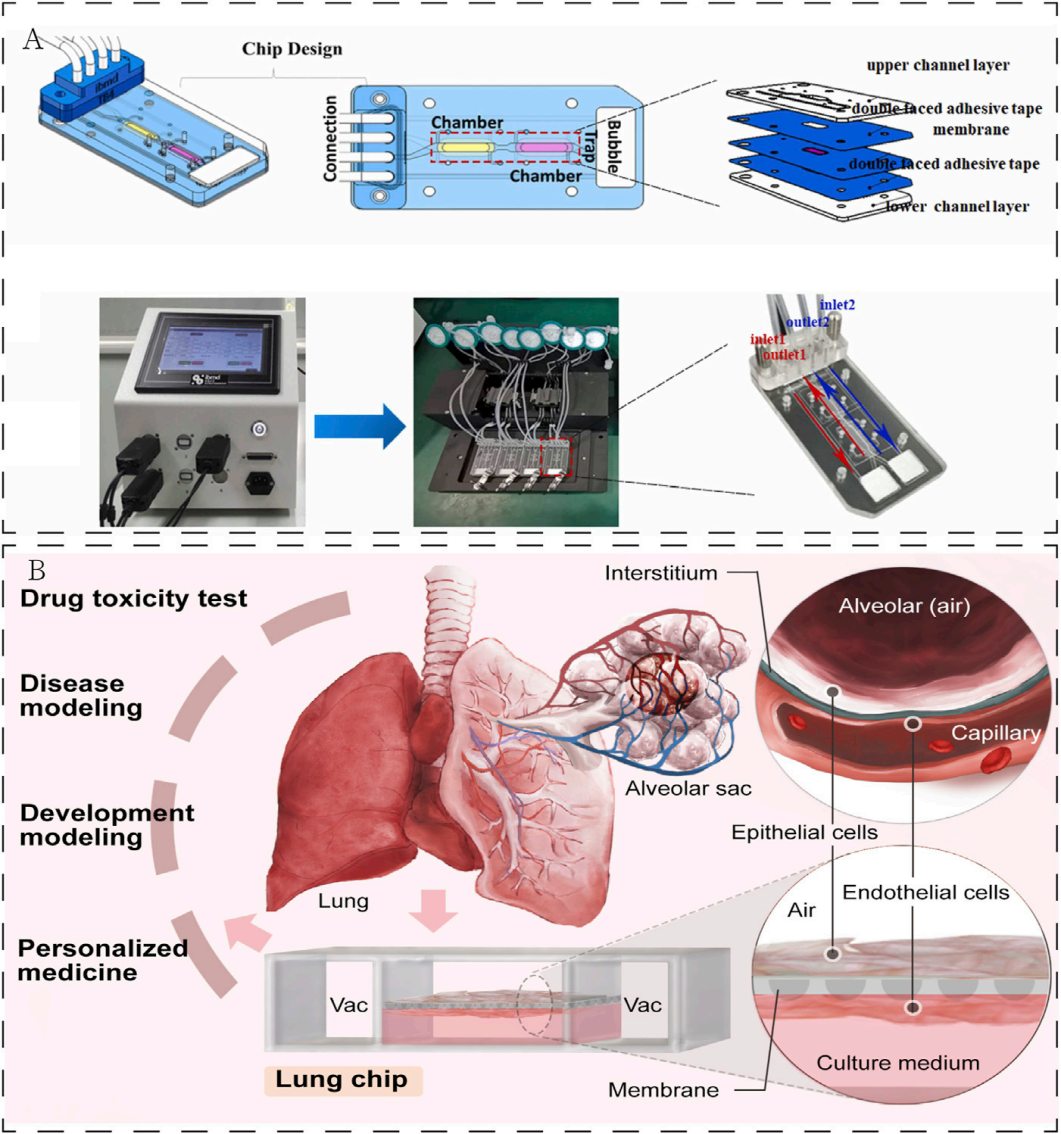
**(A)** Establishment of LOC. Reproduced with permission from Yang et al. (2023) Copyright 2023, Elsevier. **(B)** Application of LOC in the field of biomedicine.

Animal models and cancer cell lines are currently used to investigate the majority of anticancer drugs, which can provide cell-type specific mechanistic information. However, the lung is a complex organ with the specific internal environment and architecture, including alveolar-capillary barrier, extracellular matrix (ECM) and multi-organs interactions. These models have poor predictive performance for the key features to lung because it is difficult to replicate biological functions (Vlachogiannis et al., 2018), which contributes to high failure rates in new drug development (Ainslie et al., 2019; Dowden and Munro, 2019; Gao et al., 2022). The majority of experimental *in vitro* cell culture models are relatively simple. These two-dimensional models focus on understanding the molecular mechanisms of cell biology rather than integrated, complex physiological function (Kapalczynska et al., 2018). To overcome these difficulties, it is necessary to develop accurate and highly efficient modules for evaluation of anticancer drugs. In recent decades, microfluidics is an emerging tool to perform analyses with high sensitivity, speed, throughput, and low cost (Ren et al., 2013). Microfluidic organ chips, a multidisciplinary cross-product, have become one of the most valuable animal-replacement models based on such advantages as highly simulating the microenvironment *in vivo* and convenient measurement of physiological parameters (Huh et al., 2010; Benam et al., 2016). These microfluidic organ chips can reconstruct the pathophysiological features of tumours at the microscale level *in vitro*, such as the tumour microenvironment, three-dimensional tissue structure, and dynamic culture conditions, in which different types of cells are cultured on a chip according to tissue-specific three-dimensional space to simulate a specific organ or system (Baptista et al., 2022; Ingber, 2022). Briefly, the LOC contains a porous membrane and microfluidic channel system. A fluid control system that controls the fluids loading and perfusion can be connected to LOC to obtain an appropriate fluid flow to mimic the microenvironment (Figure 1A). The advent of LOC provides a powerful platform for real-time assessment of the effectiveness of drugs used in lung cancer treatment. This platform is a fast-expanding field in diagnostic, therapeutic applications, biotechnology, and drug toxicity test (Figure 1B). It enables personalized treatment of various diseases by providing efficient biological sample preparation, evaluation, and controlled distribution procedures.

## 2 Advances and challenges in the treatment of lung cancer

As the incidence of lung cancer has remained high worldwide, discovering new treatment strategies to effectively decrease the incidence and mortality of lung cancer is one of the most important tasks today (Barta et al., 2019). The development of lung cancer is a multifaceted process involving aspects such as angiogenesis, circulation, and the establishment of a tumor lesion (Inamura and Ishikawa, 2010). Lung cancer when detected are most often in a metastatic stage IV. Despite the abundance of current treatment options, the prognosis for patients remains poor (Halliday et al., 2019; Sepesi et al., 2020; Parums, 2022). For surgically resected lung cancer, careful evaluation reveals that in low-stage tumors, the neoplasm often invades surrounding tissues, leading to an increased recurrence rate and shortened patient survival (Gabor et al., 2004). In most cases, chemotherapy remains the crucial clinical strategy for the disease. However, drug resistance and drug-induced toxicity significantly hinder the effectiveness of chemotherapy (Califano et al., 2012). Currently, research on specific genes and regulatory molecules is increasing, with various targeted drugs being introduced as well (Ye et al., 2021). How to individualize and make drug administration more precise, as well as how to reduce the development of drug resistance and toxicity after use are crucial factors to address (Liu et al., 2019). In recent years, sophisticated biotechnologies and interdisciplinary integration have provided innovative approaches for the treatment of lung cancer. In the advancement of lung cancer research, LOC models has played a significant role (Table 1).

**TABLE 1:** Lung cancer studied using LOC.

Year	Cell types	Fabrication techniques	Respiratory membrane	Functions	Ref
2013	A mono-lung cancer cell line, a mixture of lung cancer, stromal cell lines, and cells from fresh lung cancer tissues	Lithography-based microfabrication techniques	Polydimethylsiloxane (PDMS) membrane	Drug sensitivity screening for better lung cancer chemotherapy regimens	Lai Benjamin et al. (2020), Xu et al. (2013)
2016	Bronchial epithelial, lung cancer, microvascular endothelial, mononuclear, and fibroblast cells	Lithography-based microfabrication techniques	PDMS membrane	Simulating the *in vivo* microenvironment of tumor metastasis and studying the cell-cell interactions during the metastatic process	Cheng et al. (2021), Xu et al. (2016)
2017	Human alveolar epithelial cells and pulmonary microvascular endothelium	Co-plating techniques	Transwell membrane	Reproduce NSCLS growth and invasion patterns; study cancer persistent cells and mechanisms of tumour dormancy *in vitro*	Si et al. (2021), Hassell et al. (2018)
2018	Human non-small cell lung cancer cells (A549) and human foetal lung fibroblasts (HFL1)	Soft lithography etching technology	Poly (lactic-co-glycolic acid) (PLGA) electrospinning nanofiber membrane	Simulate *in vitro* the tumour microenvironment alveolar biochemical factors and evaluate EGFR-targeted antitumour drug	Hassell et al. (2018), Yang et al. (2018)
2021	Human primary alveolar epithelial cells (hAEpCs) and human lung microvascular endothelial cells (VeraVec)	Lithography-based microfabrication techniques	Soft collagen–elastin (CE) membrane	Investigate basic science questions, screen compounds in drug development, model lung diseases and identify the best treatment option for each patient in precision medicine	Shrestha et al. (2020), Zamprogno et al. (2021)
2021	A549 cells, HUVECs, and human lung fibroblasts	3D printing technology	PDMS membrane	Three-dimensional vascularized lung cancer-on-a-chip model mimicking tumor microenvironment and metastasis	Ozyurt et al. (2023), Park et al. (2021)
2022	Lung cancer cells and vascular endothelial cells	3D printing technology	PDMS membrane	Testing the effects of different EGFR-targeted drugs on NCI-H650 cells and primary lung cancer cells	Ingber (2020), Tan et al. (2022b)

## 3 LOC platforms for preclinical study

*In vitro* models are the basis for clinical medicine and pharmacological research. Microfluidic and microfabrication technologies have led to rapid developments in the study of various *in vitro* models (Nikolic et al., 2018; Kohl et al., 2021). However, the majority of the current *in vivo* and *in vitro* models have many limitations, including not only differences in histiocyte physiology between animals and humans but also ethical issues of histiocyte acquisition, which makes it challenging to translate data from biological models to humans and to accurately predict human responses to drugs (Hachey and Hughes, 2018). In contrast to standard cell cultures, microfluidic chip technology, integrates the basic operation units of model preparation, reaction, separation, and detection onto a chip to perform various functions of a biological or chemical laboratory. Recently, developments in microfluidics, particularly organic microarrays, have provided powerful tools for dynamic drug activity evaluation and real-time systemic responses (Sontheimer-Phelps et al., 2019; Ingber, 2022; Tian et al., 2022).

**FIGURE 2:**
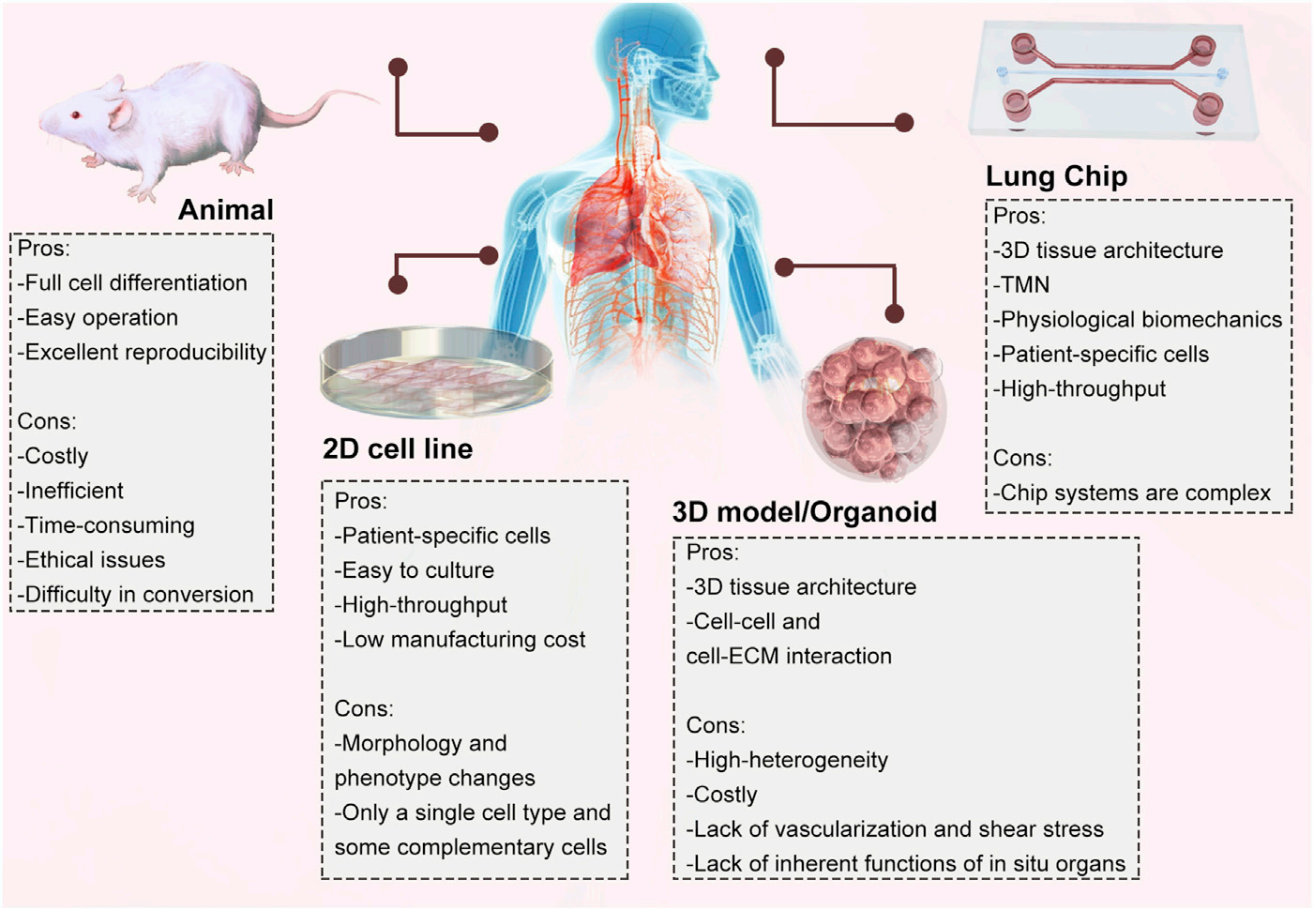
Comparison of different *in vitro* and *in vivo* models and their advantages and disadvantages.

An organ-on-a-chip is usually a microfluidic device for cell culture that comprises continuously perfused chambers populated by living cells organized to recapitulate the physiological functions of tissues. LOC was one of the first organs-on-a-chip developed, not only for modelling and drug evaluation of diseases such as pulmonary oedema, pulmonary thrombosis and lung tumours (Huh et al., 2012; Hassell et al., 2018; Jain et al., 2018; Yang et al., 2018) but also for lung diseases caused by viral infections (Ran et al., 2019; Deinhardt-Emmer et al., 2020). Huh et al. first proposed and revealed that cyclic mechanical strain exacerbates the toxic and inflammatory response of the lung to silica nanoparticles. This LOC can accurately mimic interactions between different tissues of the lung and reproduce relevant physiological functions of the lung at the alveolar level (Huh et al., 2010). The advent of LOC eliminates the disadvantage that animal models and 2D cell culture models cannot replicate the results observed *in vivo* (Francis et al., 2022). The comparison between the LOC and other existing models is illustrated (Figure 2).

### 3.1 Previous models and their limitations

#### 3.1.1 Advantages and disadvantages of animal models

Animal models are widely used for studying respiratory disease pathophysiology, sequencing the cancer genome, searching for drug targets and toxicological research. But, analyses of drug pharmacokinetics (PKs) and pharmacodynamics (PDs) performed in animals are often not predictive of drug PKs and PDs in humans (Herland et al., 2020). Currently, more complex somatic mouse models of NSCLC (Marcazzan et al., 2021; Tien et al., 2021; Chen et al., 2022) and SCLC (Gonzales et al., 2018) have been produced that can highly mimic human lung cancer, which is crucial for the study of tumour drug resistance. However, animal models are not truly representative of human physiology, pathology and genetic characteristics and therefore cannot accurately predict how a drug will respond in the human body (Ahadian et al., 2018). For example, there is a significant difference in the timing of lung development occurring between mice and humans. This different pace of lung development leads to a greater sophistication of bronchial tree branch development in humans (Metzger et al., 2008). Additionally, animal models often face many ethical issues (Liguori et al., 2017). Therefore, more accurate preclinical models for disease modelling and drug testing are needed to improve the success of new drug development and bring them to market.

#### 3.1.2 Advantages and disadvantages of 2D cell culture

2D cell culture models are still dominating preclinical evaluation of drug candidates due to their low manufacturing cost and high throughput for many biological studies (Ahadian et al., 2018; Tan C. L. et al., 2022). These models can provide a controlled and simplified environment to observe and examine cellular responses to potential therapeutic agents. Currently, in most research laboratories, the efficacy and cytotoxicity of medicines are mostly dependent on 2D cell culture systems, as this is the most convenient and low-cost method (Goodman et al., 2008). However, the main limitation of 2D cell models is that they usually consist of a single cell type and some complementary cells. These models do not replicate the complex structure and function of cells in human tissue (Jensen and Teng, 2020). Moreover, the single-culture mode can affect some physiological functions, such as cell histological morphology, cell division pattern, cell secretion, and gene expression (Kilian et al., 2010). In addition, when grown in 2D cultures, intracellular signalling pathways, for example, associated to cell proliferation, are affected by loss of cell polarity (Birgersdotter et al., 2005).

#### 3.1.3 Advantages and disadvantages of 3D cell culture/organoids

Compared to traditional 2D monolayer cultures of cells, the 3D structure of solid tumours may lead to different growth profiles and drug responses (Katt et al., 2016). 3D models for drug screening pay more attention to differences in cell shape, density and drug sensitivity (Le et al., 2016). Additionally, novel 3D cell-cultivation models encapsulate cancer biology in the microenvironment, which is a major breakthrough compared to 2D culture systems. 3D cell culture models provide a highly predictive system for precision medicine, drug screening and preclinical research (Alemany-Ribes and Semino, 2014).

Currently available 3D cancer cell models can be classified as scaffold-free spheroid 3D models, scaffold-embedded cell 3D models and microfluidics platform models (Van Zundert et al., 2020). One of the most widely used scaffold-free 3D models is the multicellular tumour sphere (MCTS) model. MCTSs can be formed by proliferation of individual cells into cell aggregates or by further proliferation of preaggregated cell clusters (Baraniak and McDevitt, 2012). 3D single-cell models can well simulate tumour morphology, function, and microhabitat *in vivo* (Liao et al., 2019). For embedded models, there are usually two categories: natural and synthetic. The most commonly used natural scaffolds are composed of collagen, elastin, gelatine, and hyaluronic acid polymer matrix or substrate (Hughes et al., 2010). This model is more similar to the microenvironment but still has the disadvantages of no perfusion, no stress, and limited vasculature. The other natural scaffold construct is taken from mouse sarcoma cells and closely resembles the physiological extracellular matrix (ECM), containing a mixture of ECM proteins, including collagen, fibronectin and laminin. With development of microfluidics platforms, microfluidic technology that allows fluids to pass through the system to deliver nutrients and remove cellular waste by pump or gravity has been used in a preclinical model (Low and Tagle, 2017). This technique allows for a good representation of the spatial structure and distribution within tumour cells, which can reveal the process of proliferation, migration and invasion of tumour cells and their interactions with each other (Holton et al., 2017). However, low spheroid quantities, difficulty in renewing the culture medium, high cost, and the need for large initial volumes and long periods of time for cancer cell growth limit its widespread use (Sachs et al., 2018; Vlachogiannis et al., 2018; Zhang et al., 2018). A previous study showed that spheroids composed of patient-derived tumour cells could maintain tumour biology *in vitro* for a long period of time (Song et al., 2018). All these results suggest that spheroid culture has important applications in the screening of new anticancer drugs. However, *in vitro* cell culture studies are limited and do not fully mimic the more complex *in vivo* tumorigenesis and drug response to tumour therapy.

### 3.2 Several LOC models prepared using different technologies

#### 3.2.1 Characteristics of LOC manufactured using lithography-based microfabrication techniques

**FIGURE 3:**
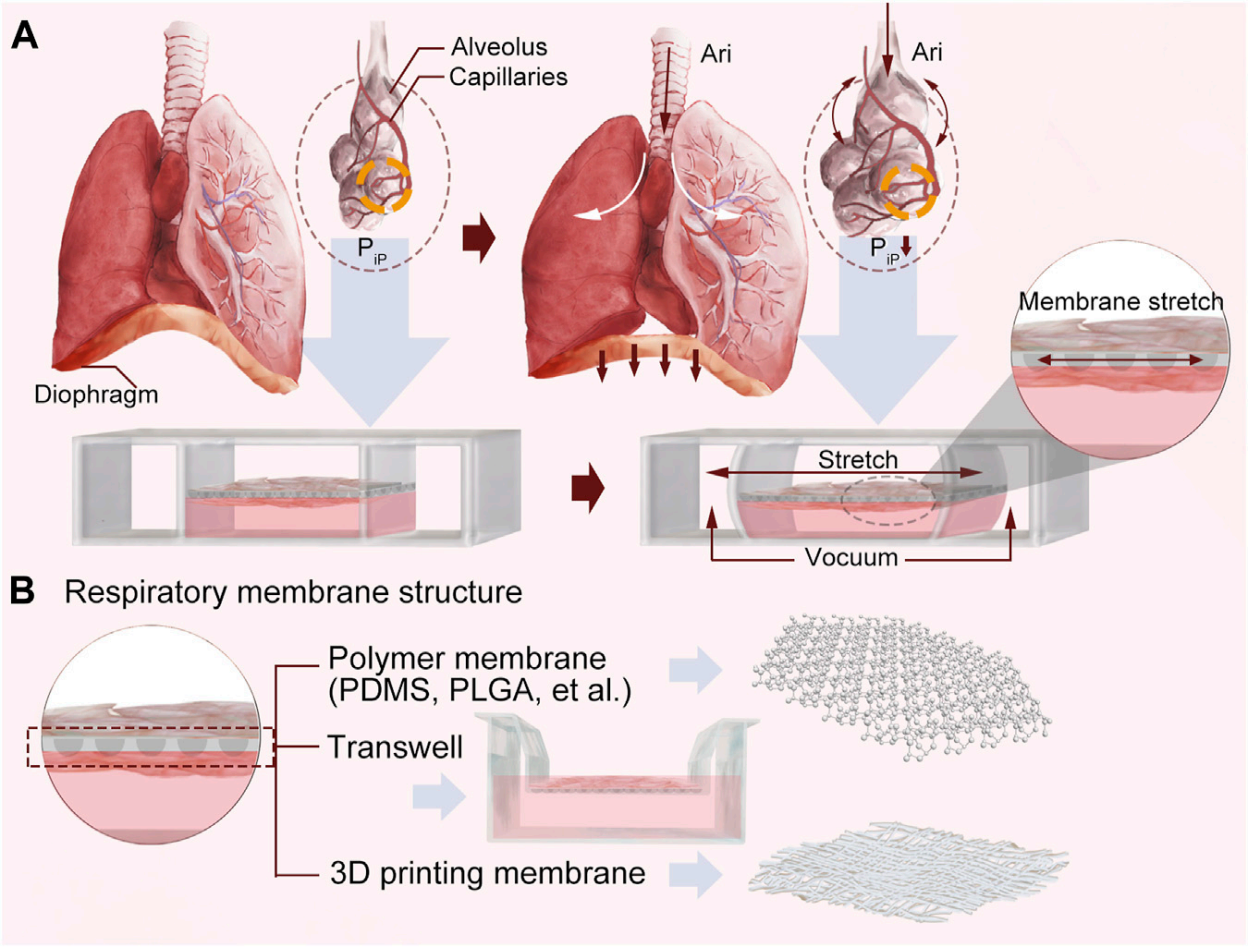
**(A)** A schematic diagram of the principle. **(B)** Respiratory membrane structures designed using three different technologies.

Huh et al. (2010) designed a polymer polydimethylsiloxane (PDMS) chip via soft lithography-based microfabrication techniques (Figure 3B). This model was fabricated by microfabricating a microfluidic system containing two closely arrayed microchannels separated by a thin (10 µm) porous flexible membrane of PDMS. The porous membrane was coated with ECM (fibronectin or collagen), and cultured human alveolar epithelial cells and human pulmonary microvascular endothelium were placed on opposite sides. Once alveolar cell growth in the epithelial compartment of the porous membrane formed tightly connected junctions, the epithelial compartment media were aspirated to create an air-fluid interface. The flow of the culture medium in the endothelial compartment was maintained. By varying the pressure in the vacuum cavity on both sides of the chip, the membrane was deformed in a regular elastic manner to simulate breathing movements (Figure 3A). Though beneficial for drug and toxicity testing, the device cannot completely replicate organ-level lung functions. Sellgren et al. (2014) designed a new LOC model of a dual-membrane integrated microfluidic device based on previous microarrays. A microfluidic model of three vertically stacked chambers separated by a PDMS porous membrane was developed to simulate the microstructure of the airway mucosa. The airway microstructural properties were well simulated through three interfaces: the air-liquid interface, lung fibroblasts and microvascular endothelial cells. This LOC demonstrates that a microfluidic chip can support culture of primary airway epithelial cells and illustrates a co-culture approach enabling heterotypic cell interaction while maintaining compartmentalization.

#### 3.2.2 Characteristics of LOC manufactured using thermoplastic techniques

Humayun et al. (2018) developed a plastic airway-on-a-chip airway device using micromilling and solvent bonding to create a thermoplastic-based LOC model by using suspension hydrogels instead of PDMS to separate the upper and lower microchannels (Figure 3B). The chip contained an epithelial cell (EC) layer, a suspended hydrogel layer and a smooth muscle cell (SMC) layer, and all three microchannels were stacked vertically on top of each other. Unique to the design is that the upper and lower interfaces of the suspension hydrogel are used for EC and SMC cultures, respectively, and that the hydrogel can be extracted for further study, helping to investigate interactions between ECs, SMCs and the cell matrix in the development of lung cancer.

#### 3.2.3 Characteristics of LOC manufactured using 3D cell bioprinting

The assistance of 3D bioprinting in tissue modeling and the fabrication of organ-like structures has fostered the development of LOC, with a wide range of applications, including personalized medicine, drug delivery, therapeutics, and many more. Park et al. (2018) constructed an alveolar-capillary interface in the human lung, and an LOC chip with two opposing microchannels was developed using 3D cell bioprinting technology containing a vascular network (Figure 3B). To simulate an airway microenvironment with functional capillaries, decellularized ECM (d-ECM) isolated from various organs/tissues was used as a bioink. A vascular platform (VP) was developed by direct 3D printing of cell-loaded d-ECM bioinks. The chip has a central reservoir for EC bioink and two reservoirs for lung fibroblast (LF) bioink on each side to regulate medium flow. This LOC, manufactured by 3D cell printing technology, allowed for independent control and variation in the precision of the parameters and efficient integration with the vascular network platform to reproduce the structural function of the organ.

## 4 Progress in LOC in lung cancer

Drug resistance has always been a major obstacle in treatment of lung cancer (Shanker et al., 2010; Lim and Ma, 2019), and previously introduced models do not fully show the complexity of the tumour microenvironment. Organ-on-a-chip is able to simulate the *in vivo* tumour biology environment effectively and therefore has received increasing attention from scholars and inspired them to conduct in-depth research (Nawroth et al., 2019; Guzzeloni et al., 2022). The LOC model allows for studying drug resistance in the context of the lung microenvironment, including effects of factors such as fluid flow, oxygen concentration, and cell‒cell interactions. One of the key advantages of LOC technology is its ability to accurately replicate the *in vivo* lung environment, which can lead to highly accurate and reliable results. The chip also provides the ability to rapidly screen drugs and evaluate their effectiveness in a high-throughput manner, making it a valuable tool for drug discovery and development.

### 4.1 The tumour microenvironment for drug resistance mechanism

The tumour microenvironment (TME) is not recapitulated in previous models used in cancer investigation, limiting the translation of preliminary findings to clinical practice. The TME is a complex and diverse multicellular environment. It is usually composed of immune cells, endothelial cells, ECM and other secreted molecules, such as growth factors and cytokines, as well as blood and lymphatic vessels, which are collectively enmeshed with each other and with the heterogeneous cancer cells (Bejarano et al., 2021; Wu et al., 2021; de Visser and Joyce, 2023). Tumour tissues usually have specific microenvironments that differ from normal tissues, such as acidity, hypoxia, and enzyme overexpression (Shrivastava et al., 2004). Additionally, tumour cells can affect the immune system by producing immunosuppressive TME, promoting immune evasion and ultimately leading to drug resistance. Decreased immunity of the body to tumour cells leads to immune editing to produce a microenvironment that promotes tumour growth, such as increased tumour-associated macrophages (TAMs) and regulatory T cells, decreased absolute counts of lymphocytes, and apoptosis of cytotoxic T cells (Anichini et al., 2020; Kunimasa and Goto, 2020; Zhang et al., 2022).

However, the TME involves not only tumorigenesis, metabolism and metastasis but also highly structured spatial structures and the relationship between cancer cells and surrounding nonmalignant cells (Wlodkowic and Cooper, 2010). This interaction between cells affects stromal development and ultimately the efficacy and efflux of anticancer drugs (Lai Benjamin et al., 2020). LOC can reconstruct artificial airways on a chip to precisely control drug diffusion rates, fluid shear stress, and even microscale cellular niches (Walsh et al., 2009). It serves as a novel 3D microfluidic culture platform that reconstructs the connection between tumour cells and surrounding tissue cells and mimics the physiological structure of normal human lung tissue. Lai et al. (2020) designed a model that can remodel tumour stiffness through myofibroblast contraction and collagen deposition. This study illustrated that the efficacy of antitumour drugs is affected when the TME changes. Therefore, the structural organization of tumour cells within tumour tissue determines how these cells function and interact in their surrounding environment. Xu et al. (2013) constructed a three-dimensional coculture drug sensitivity assay chip based on microfluidic technology. The sensitivity of antitumour drugs was detected by reconstructing the *in vivo* tumour microenvironment. The drug concentration that was most sensitive to tumour cells was screened according to the concentration gradient generator (CGG) within the chip to guide individualized treatment of lung cancer. In addition, Russell Jenkins and others adapted a 3D microfluidic device to culture patient-derived organotypic tumor spheroids, preserving the immune architecture of the tumor microenvironment, and enabling real-time analysis (Jenkins et al., 2018). This *ex vivo* system accelerates the identification of novel predictive and/or prognostic biomarkers, contributing to precise treatment of tumors. Recently, Benam et al. (Vo et al., 2024) constructed user-controlled long rounded EMC embedded vascular microlumens on-chip, demonstrating the significant influence of microchannel cross-sectional geometry and length on the uniform distribution and magnitude of shear stress on the vascular wall. These advantages render LOC particularly useful in various applications including disease modeling.

### 4.2 High-throughput analysis and deep learning on chips

The high-throughput and deep learning technology evaluation function of the LOC model also provide great convenience for development and screening of novel antitumour drugs in disease models (Ko et al., 2018; Kim et al., 2022). Microfluidic technology allows for precise and efficient adjustment of a device measuring a few square centimetres (or smaller), optimizing conventional chemical and biological laboratory operations, simplifying experimental procedures, and saving time and labour costs. This may greatly promote development of drug research (Azizgolshani et al., 2021). The integration of multiple devices into a single platform, especially in cancer and metastatic studies for various analyses, results in a high throughput system that generates amounts of intricately intertwined data (Oliver et al., 2019). Sebastiaan et al. (Trietsch et al., 2017) conducted an experiment with a gut-on-a-chip containing 357 gut tubes, generating 20,000 data points, which represents the largest published Organ-on-a-Chip dataset to date. Deep learning involves a range of computational methods extensively employed across various domains to reduce large numbers of measurements into lower-dimensional outputs that are more useful (Kourou et al., 2015). Furthermore, the applications of deep learning in microfluidic chips have allowed researchers to observe phenomena that were difficult to capture in the past. Singh et al. (2017) using the aforementioned approach to identify each cell flowing through the microchannel at a rate of 10,000 cells per second. This approach achieved label-free cell classification and enabled rapid identification of tumor cells. However, the design and modeling of microfluidic chips require a substantial background in computation and fluid mechanics, which limits the widespread application and dissemination of this technology.

### 4.3 Screening for drug-resistant cancer cells

Screening for cancer cells carrying mutations in drug resistance-related genes is of great value in early diagnosis and treatment of lung cancer. Detection and screening of anticancer drug-resistant cancer cells harbouring genes with single-nucleotide mutations has received much attention in cancer diagnosis in recent years (Xu et al., 2013; Sander et al., 2019; Shigeto et al., 2020). Shigeto et al. (2020) used single-cell microarray chips and peptide nucleic acid (PNA)-DNA probes to specifically detect T790M mutant cancer cells. Among secondary drug resistance mutations, EGFR-mutant phenotypes are most common in NSCLC due to mutations in the receptor-binding region of the EGFR gene (T790M) driving secondary mutations in the kinase domain, reducing the affinity of tumour cells for targeted therapies (Sun et al., 2022; Zhou et al., 2022). Tyrosine kinase inhibitors (TKIs), as represented by gefitinib, are molecularly targeted anticancer drugs that bind to the structural domain of the EGFR protein tyrosine kinase (Lynch et al., 2004; Yun et al., 2008), inducing cell death by inhibiting the signal transduction of epidermal growth factor (Kobayashi and Mitsudomi, 2016). Therefore, analysis of the proportion or number of cancer cells comprising the T790M mutation is essential for drug-resistant NSCLC. The results showed that cancer cells with the T790M mutation had a strong fluorescent signal, which provided an effective pathway for early diagnosis of NSCLC. Yang et al. (2018) used electrospinning technology to develop an LOC with a poly (lactic-co-glycolic acid) (PLGA) membrane. In addition, cultured lung cancer cells, lung fibroblasts and vascular endothelial cells on both sides of the membrane were used to evaluate the antitumour effect of gefitinib. These factors have made LOC a promising drug screening tool, providing a scientific basis for its widespread use.

### 4.4 Auxiliary application of NDDS in LOC

Due to various biological barriers in the human body, traditional drug delivery methods are often inefficient, have nonspecific distribution, and have difficulty reaching the target site (Blanco et al., 2015). Development of NDDSs for reversing drug resistance offers a novel strategy for tumour treatment (Calixto et al., 2016; Majidinia et al., 2020). The current NDDSs used for clinical treatment mainly include organic NDDSs (Huang et al., 2018; Jain and Jain, 2018), inorganic NDDSs (Yang and Yu, 2016), and composite multifunctional NDDSs (Liu et al., 2021). These nanodrug carriers have a strong drug-carrying capacity to transport drugs to specific targets to improve drug efficacy (Liu et al., 2017; Moosavian and Sahebkar, 2019). Most antitumour drugs are small molecule compounds with poor water solubility, low drug metabolism kinetics and other defects (Ke et al., 2018). Because of the lack of tissue selectivity and targeting, they are widely distributed to various tissue sites after entering the circulatory system and are easily captured and metabolized by the reticuloendothelial system (RES) or excreted through glomerular filtration (Kobayashi et al., 2014; Shirali and Sprangers, 2022). This not only reduces the utilization rate of drugs but also increases their toxic side effects. Currently, nanotechnology, especially nanopreparation technology, is used to combine drugs with inorganic or organic materials in certain ways (including chemical bonding, physical embedding, encapsulation, electrostatic adsorption, etc.) and control the diameter to 0.1–100 µm to prepare nanomedicines (De Jong and Borm, 2008). When nanomedicines enter the circulatory system, due to their unique high permeability and long-term retention (EPR) effect, they can enhance drug accumulation in tumour tissue and slow down drug efflux (He et al., 2015; Park et al., 2019; Shi et al., 2020). In addition, some nanomaterials have special properties, such as temperature-sensitive properties (Amin et al., 2020) and pH-sensitive properties (Fang et al., 2020). These strategies provide broad prospects for drug release in specific environments.

Many potential chemotherapeutic agents are not available for clinical use due to off-target toxicity, unstable metabolism or poor pharmacokinetics. NDDS provides drugs in a controlled and specific manner, offering feasible solutions for drug development to address the high clinical failure rate. Nanodrug delivery systems (NDDSs), such as nanoparticles and nanocapsules, allow for antineoplastic drugs to be administered to patients while modulating the location and concentration of release in the body, improving drug efficacy, minimizing the exposure of healthy cells to the drug and reducing the risk of drug toxicity (Li et al., 2019). NDDSs generally use intravenous infusion to inject drugs into the circulatory system, and nanoparticles interact with dynamic blood flow; in contrast, traditional models have difficulty reproducing the hydrodynamic characteristics of blood flow (Cho et al., 2011). In addition, the interaction between nanoparticles and endothelial cells in dynamic blood is often overlooked (Sindhwani et al., 2020). However, microfluidic LOC platforms with vascular endothelial cells can dynamically evaluate the role of nanoparticles in the circulatory system. Caballero et al. (Caballero et al., 2017) described how microfluidic chips of tumor blood vessels can be used to better elucidate the behavior of new nanocarriers in the microcirculation of both healthy and cancerous tissues. They investigated phenomena such as extravasation, immune response, and endothelial targeting under flow in capillaries, which can be accurately modeled using microfluidics. This plays a crucial role in advancing next-generation targeted drug delivery methods.

### 4.5 Toxicology research

Drug-induced toxicity is one of the most important issues resulting from inaccurate preclinical models (Dzidic-Krivic et al., 2024). For example, approximately 20% of acute kidney injuries (AKI) after hospitalization can be attributed to nephrotoxicity induced by pharmaceutical agents (Kim and Moon, 2012). There is an urgent need for more precise and accurate techniques to reconstruct organ-specific therapeutic features associated with drug interventions, including examining drug delivery and real-time monitoring of cellular and tissue responses to specific medications. Organ-on-a-Chip technology has the potential to improve success rate of drug development pipelines, as it can recapitulate organ-level pathophysiology and clinical responses. Ewart et al. (Ewart et al., 2023) analyzed 870 liver chips to assess their ability to predict drug-induced liver injury. The results showed that the liver chips achieved a sensitivity of 87% and a specificity of 100% through blind testing with 27 known hepatotoxic and non-toxic drugs. Ingber et al. (Goyal et al., 2024) also utilized human organ chips to conduct research in regenerative pharmacology and toxicology, revealing healing and regeneration mechanisms in various human tissues, which provides crucial insights for predicting the clinical efficacy or toxicity of drugs. Therefore, integrating predictive organ chips into the drug development can significantly enhance drug discovery and development, allowing manufacturers to bring safer and more effective drugs to market in less time and at lower costs.

### 4.6 Personalized medicine

In clinical settings, the efficacy and tolerability of drugs vary significantly among populations. There are individual differences among different lung cancer patients, and variations in sensitivity to various targeted drugs exist. For example, when T790M mutation or deletion occurs, the use of EGFR TKIs in treatment is more likely to result in decreased affinity and drug resistance (Meador and Hata, 2020). Currently, genomic sequencing of solid tumors and identification of key molecular targets have fundamentally altered the treatment approach for both primary and metastatic cancers, turning them from fatal diseases into chronic conditions, and even achieving complete cures. Benam et al. (2016) designed a human lung ‘small airway-on-a-chip’ for the analysis of organ-level lung pathophysiology *in vitro*. Exposing patient epithelial cells to Interleukin 13 (IL-13) simulates the effects of asthma exacerbation. By obtaining patient-specific stem cells to reconstruct the inflammatory phenotype on-chip, not only can the efficacy of novel experimental therapies be tested, but also the molecular mechanisms of drug action can be dissected at the level of *in vitro* human organ backgrounds. Veith et al. (2024) employed patient-derived lung tumor chips to assess personalized responses to anti-PD-1 therapy. By harnessing the power of real-time imaging and advanced image analysis algorithms, they rapidly and accurately measured the impact of immune checkpoint inhibitors on T cell-mediated cancer cell death, paving the way for a new approach to analyzing personalized immune therapy responses. However, personalized medical applications on chips still face many limitations. Certain cell types, such as cardiomyocytes and neurons, are difficult to maintain regeneration and differentiation *in vitro* environments (Zhang et al., 2017). These cultures are still evidently far from fully recapitulating the complexity of the *in vivo* situation. Therefore, the potential predictive power of such a chip approach could not be validated. Larger patient cohorts and biopsy-compatible chips will be necessary.

### 4.7 Effect of ferroptosis on drug resistance

Ferroptosis is a newly discovered form of immunogenic cell death (ICD). It is driven by iron-dependent phospholipid peroxidation and regulated by multiple metabolic and signaling pathways (Stockwell, 2022). Ferroptosis is typically accompanied by the abundant accumulation of intracellular iron and the generation of oxidative hydroxyl radicals. It is characterized by the production of lethal levels of iron-dependent lipid peroxidation (Chen et al., 2021; Wang D. et al., 2023). Recent research has shown that ferroptosis in tumour cells may cause robust antitumour immune effects, even in drug-resistant cancer types (Wang et al., 2020). Dai et al. (Wang et al., 2021) developed an assembly of exosome inhibitor (GW4869) and ferroptosis inducer (Fe3+), aimed at reversing exosome-mediated immune suppression by programmed death-ligand 1(PD-L1) and enhancing tumor ferroptosis, thereby improving the efficacy of immunotherapy against tumors. Some recent studies have found that ferroptosis is also associated with various types of lung injury. For example, Fan et al. found that PM2.5-mediated ferroptosis causes endothelial cell damage in human lungs (Guohua et al., 2021). Toxic PM2.5 can invade the small airways and interfere with lung physiology, eventually leading to chronic lung damage and even lung cancer (Cheng et al., 2021). Alvarez et al. reported that the protein NFS1 protects lung cancer cells from ferroptosis. When blocking NFS1, high oxygen levels can lead to degradation of iron–sulfur clusters in lung cancer cells, thereby delaying their growth and making them easier to kill. Therefore, constructing a lung cancer model using lung chips can simulate a high oxygen environment to avoid ferroptosis caused by ROS. Such efforts may lead to a new strategy for ferroptosis-mediated anticancer therapy.

## 5 Conclusion and remarks

LOC is an emerging technology based on microfluidic platforms and *in vitro* cell culture. Numerous studies have revealed the broad prospects of LOC for diagnosis and treatment of respiratory diseases (Shrestha et al., 2020; Si et al., 2021). It can reconstruct the microstructure of the alveolar capillary unit on a microfluidic chip, simulate the alveolar air‒water interface, accurately manipulate the tumour microenvironment, and analyse the effects of shear force, tension, and pressure on various physiological and pathological lung functions. Such analysis of physical and microenvironmental factors on cells and tissues cannot be achieved by other *in vitro* models.

Although organ chips have some advantages compared to other *in vitro* models, they do have certain limitations. First, most LOC devices use PDMS copy moulding methods. However, PDMS may absorb a portion of hydrophobic drugs, which may affect the accuracy of evaluating concentrated drug solutions in high-surface microchannels (Ozyurt et al., 2023). Therefore, researchers are actively looking for alternatives or new technologies for PDMS, such as hydrogels, thermoplastic materials, and 3D printing materials. Second, it is important to mention that while researchers can currently mimic the important characteristics of cancer physiology using *in vitro* 3D tumour models, even the most advanced 3D cancer models cannot fully mimic tumour physiology and, therefore, cannot fully replace animal models in drug delivery research (Ingber, 2020). Furthermore, the study of inter-organ communication is presently unattainable, multi-organ-a-chip can also be considered to solve such limitation.

In this review, we introduced LOC models and their achievements in lung cancer and drug development. LOC models provided an efficient and accurate platform for modelling lung diseases as well as antitumour drug studies. These systems can reduce the cost of medication development, lessen the social and economic burden of lung cancer, and decrease the reliance of researchers on conventional *in vitro* and animal models. Moreover, the combination of LOC and emerging technologies not only facilitates drug discovery and development but also contributes significantly to development of translational medicine and toxicology. We believe that organ-on-a-chip technology will bring more revolutionary changes to others in the field in the future.

## References

[B1] Ahadian S. Civitarese R. Bannerman D. Mohammadi M. H. Lu R. Wang E. (2018). Organ-on-A-chip platforms: a convergence of advanced materials, cells, and microscale technologies. Adv. Healthc. Mater 7. 10.1002/adhm.201800734 29034591

[B2] Ainslie G. R. Davis M. Ewart L. Lieberman L. A. Rowlands D. J. Thorley A. J. (2019). Microphysiological lung models to evaluate the safety of new pharmaceutical modalities: a biopharmaceutical perspective. Lab. Chip 19, 3152–3161. 10.1039/c9lc00492k 31469131

[B3] Alemany-Ribes M. Semino C. E. (2014). Bioengineering 3D environments for cancer models. Adv. Drug Deliv. Rev. 79-80, 40–49. 10.1016/j.addr.2014.06.004 24996134

[B4] Amin M. Huang W. Seynhaeve A. L. B. Ten Hagen T. L. M. (2020). Hyperthermia and temperature-sensitive nanomaterials for spatiotemporal drug delivery to solid tumors. Pharmaceutics 12, 1007. 10.3390/pharmaceutics12111007 33105816 PMC7690578

[B5] Anichini A. Perotti V. E. Sgambelluri F. Mortarini R. (2020). Immune escape mechanisms in non small cell lung cancer. Cancers (Basel) 12, 3605. 10.3390/cancers12123605 33276569 PMC7761620

[B6] Atmaca H. Oguz F. Ilhan S. (2022). Drug delivery systems for cancer treatment: a review of marine-derived polysaccharides. Curr. Pharm. Des. 28, 1031–1045. 10.2174/1381612828666220211153931 35152862

[B7] Azizgolshani H. Coppeta J. R. Vedula E. M. Marr E. E. Cain B. P. Luu R. J. (2021). High-throughput organ-on-chip platform with integrated programmable fluid flow and real-time sensing for complex tissue models in drug development workflows. Lab. Chip 21, 1454–1474. 10.1039/d1lc00067e 33881130

[B8] Baptista D. Moreira Teixeira L. Barata D. Tahmasebi Birgani Z. King J. van Riet S. (2022). 3D lung-on-chip model based on biomimetically microcurved culture membranes. ACS Biomater. Sci. Eng. 8, 2684–2699. 10.1021/acsbiomaterials.1c01463 35502997 PMC9198974

[B9] Baraniak P. R. McDevitt T. C. (2012). Scaffold-free culture of mesenchymal stem cell spheroids in suspension preserves multilineage potential. Cell Tissue Res. 347, 701–711. 10.1007/s00441-011-1215-5 21833761 PMC4149251

[B10] Barta J. A. Powell C. A. Wisnivesky J. P. (2019). Global epidemiology of lung cancer. Ann. Glob. Health 85, 8. 10.5334/aogh.2419 30741509 PMC6724220

[B11] Bejarano L. Jordao M. J. C. Joyce J. A. (2021). Therapeutic targeting of the tumor microenvironment. Cancer Discov. 11, 933–959. 10.1158/2159-8290.cd-20-1808 33811125

[B12] Benam K. H. Villenave R. Lucchesi C. Varone A. Hubeau C. Lee H. H. (2016). Small airway-on-a-chip enables analysis of human lung inflammation and drug responses *in vitro* . Nat. Methods 13, 151–157. 10.1038/nmeth.3697 26689262

[B13] Birgersdotter A. Sandberg R. Ernberg I. (2005). Gene expression perturbation *in vitro*--a growing case for three-dimensional (3D) culture systems. Semin. Cancer Biol. 15, 405–412. 10.1016/j.semcancer.2005.06.009 16055341

[B14] Bjornmalm M. Thurecht K. J. Michael M. Scott A. M. Caruso F. (2017). Bridging bio-nano science and cancer nanomedicine. ACS Nano 11, 9594–9613. 10.1021/acsnano.7b04855 28926225

[B15] Blanco E. Shen H. Ferrari M. (2015). Principles of nanoparticle design for overcoming biological barriers to drug delivery. Nat. Biotechnol. 33, 941–951. 10.1038/nbt.3330 26348965 PMC4978509

[B16] Briolay T. Petithomme T. Fouet M. Nguyen-Pham N. Blanquart C. Boisgerault N. (2021). Delivery of cancer therapies by synthetic and bio-inspired nanovectors. Mol. Cancer 20, 55. 10.1186/s12943-021-01346-2 33761944 PMC7987750

[B17] Caballero D. Blackburn S. M. de Pablo M. Samitier J. Albertazzi L. (2017). Tumour-vessel-on-a-chip models for drug delivery. Lab. Chip 17, 3760–3771. 10.1039/c7lc00574a 28861562

[B18] Califano R. Abidin A. Z. Peck R. Faivre-Finn C. Lorigan P. (2012). Management of small cell lung cancer: recent developments for optimal care. Drugs 72, 471–490. 10.2165/11597640-000000000-00000 22356287

[B19] Calixto G. M. Bernegossi J. de Freitas L. M. Fontana C. R. Chorilli M. (2016). Nanotechnology-based drug delivery systems for photodynamic therapy of cancer: a review. Molecules 21, 342. 10.3390/molecules21030342 26978341 PMC6274468

[B20] Chen H. Zheng M. Zhang W. Long Y. Xu Y. Yuan M. (2022). Research status of mouse models for non-small-cell lung cancer (NSCLC) and antitumor therapy of traditional Chinese medicine (TCM) in mouse models. Evid. Based Complement. Altern. Med. 2022, 1–13. 10.1155/2022/6404853 PMC9519343 36185084

[B21] Chen X. Li J. Kang R. Klionsky D. J. Tang D. (2021). Ferroptosis: machinery and regulation. Autophagy 17, 2054–2081. 10.1080/15548627.2020.1810918 32804006 PMC8496712

[B22] Cheng W. Lu J. Wang B. Sun L. Zhu B. Zhou F. (2021). Inhibition of inflammation-induced injury and cell migration by coelonin and militarine in PM(2.5)-exposed human lung alveolar epithelial A549 cells. Eur. J. Pharmacol. 896, 173931. 10.1016/j.ejphar.2021.173931 33549578

[B23] Cho E. C. Zhang Q. Xia Y. N. (2011). The effect of sedimentation and diffusion on cellular uptake of gold nanoparticles. Nat. Nanotechnol. 6, 385–391. 10.1038/nnano.2011.58 21516092 PMC3227810

[B24] Deinhardt-Emmer S. Rennert K. Schicke E. Cseresnyes Z. Windolph M. Nietzsche S. (2020). Co-infection with *Staphylococcus aureus* after primary influenza virus infection leads to damage of the endothelium in a human alveolus-on-a-chip model. Biofabrication 12, 025012. 10.1088/1758-5090/ab7073 31994489

[B25] De Jong W. H. Borm P. J. (2008). Drug delivery and nanoparticles:applications and hazards. Int. J. Nanomedicine 3, 133–149. 10.2147/ijn.s596 18686775 PMC2527668

[B26] de Visser K. E. Joyce J. A. (2023). The evolving tumor microenvironment: from cancer initiation to metastatic outgrowth. Cancer Cell 41, 374–403. 10.1016/j.ccell.2023.02.016 36917948

[B27] Dowden H. Munro J. (2019). Trends in clinical success rates and therapeutic focus. Nat. Rev. Drug Discov. 18, 495–496. 10.1038/d41573-019-00074-z 31267067

[B28] Dzidic-Krivic A. Sher E. K. Kusturica J. Farhat E. K. Nawaz A. Sher F. (2024). Unveiling drug induced nephrotoxicity using novel biomarkers and cutting-edge preventive strategies. Chem. Biol. Interact. 388, 110838. 10.1016/j.cbi.2023.110838 38104745

[B29] Ewart L. Apostolou A. Briggs S. A. Carman C. V. Chaff J. T. Heng A. R. (2023). Author Correction: performance assessment and economic analysis of a human Liver-Chip for predictive toxicology. Commun. Med. (Lond) 3, 16. 10.1038/s43856-023-00249-1 36732600 PMC9894928

[B30] Fang Z. Pan S. Gao P. Sheng H. Li L. Shi L. (2020). Stimuli-responsive charge-reversal nano drug delivery system: the promising targeted carriers for tumor therapy. Int. J. Pharm. 575, 118841. 10.1016/j.ijpharm.2019.118841 31812795

[B31] Francis I. Shrestha J. Paudel K. R. Hansbro P. M. Warkiani M. E. Saha S. C. (2022). Recent advances in lung-on-a-chip models. Drug Discov. Today 27, 2593–2602. 10.1016/j.drudis.2022.06.004 35724916

[B32] Gabor S. Renner H. Popper H. Anegg U. Sankin O. Matzi V. (2004). Invasion of blood vessels as significant prognostic factor in radically resected T1-3N0M0 non-small-cell lung cancer. Eur. J. Cardiothorac. Surg. 25, 439–442. 10.1016/j.ejcts.2003.11.033 15019675

[B33] Gao W. Hu H. Dai L. He M. Yuan H. Zhang H. (2022). Structuretissue exposure/selectivity relationship (STR) correlates with clinical efficacy/safety. Acta Pharm. Sin. B 12, 2462–2478. 10.1016/j.apsb.2022.02.015 35646532 PMC9136610

[B34] Gonzales J. Kossatz S. Roberts S. Pirovano G. Brand C. Perez-Medina C. (2018). Nanoemulsion-based delivery of fluorescent PARP inhibitors in mouse models of small cell lung cancer. Bioconjug Chem. 29, 3776–3782. 10.1021/acs.bioconjchem.8b00640 30354077 PMC6548450

[B35] Goodman T. T. Ng C. P. Pun S. H. (2008). 3-D tissue culture systems for the evaluation and optimization of nanoparticle-based drug carriers. Bioconjug Chem. 19, 1951–1959. 10.1021/bc800233a 18788773 PMC2652657

[B36] Goyal G. Belgur C. Ingber D. E. (2024). Human organ chips for regenerative pharmacology. Pharmacol. Res. Perspect. 12, e01159. 10.1002/prp2.1159 38149766 PMC10751726

[B37] Guohua F. Tieyuan Z. Xinping M. Juan X. (2021). Melatonin protects against PM2.5-induced lung injury by inhibiting ferroptosis of lung epithelial cells in a Nrf2-dependent manner. Ecotoxicol. Environ. Saf. 223, 112588. 10.1016/j.ecoenv.2021.112588 34364124

[B38] Gurkan U. A. Wood D. K. Carranza D. Herbertson L. H. Diamond S. L. Du E. (2024). Next generation microfluidics: fulfilling the promise of lab-on-a-chip technologies. Lab. Chip 24, 1867–1874. 10.1039/d3lc00796k 38487919 PMC10964744

[B39] Guzzeloni V. Veschini L. Pedica F. Ferrero E. Ferrarini M. (2022). 3D models as a tool to assess the anti-tumor efficacy of therapeutic antibodies: advantages and limitations. Antibodies (Basel) 11, 46. 10.3390/antib11030046 35892706 PMC9326665

[B40] Hachey S. J. Hughes C. C. W. (2018). Applications of tumor chip technology. Lab. Chip 18, 2893–2912. 10.1039/c8lc00330k 30156248 PMC6207449

[B41] Halliday P. R. Blakely C. M. Bivona T. G. (2019). Emerging targeted therapies for the treatment of non-small cell lung cancer. Curr. Oncol. Rep. 21, 21. 10.1007/s11912-019-0770-x 30806814

[B42] Hassell B. A. Goyal G. Lee E. Sontheimer-Phelps A. Levy O. Chen C. S. (2018). Human organ chip models recapitulate orthotopic lung cancer growth, therapeutic responses, and tumor dormancy *in vitro* . Cell Rep. 23, 3698. 10.1016/j.celrep.2018.06.028 29925009

[B43] He C. Liu D. Lin W. (2015). Self-assembled core-shell nanoparticles for combined chemotherapy and photodynamic therapy of resistant head and neck cancers. ACS Nano 9, 991–1003. 10.1021/nn506963h 25559017

[B44] Herland A. Maoz B. M. Das D. Somayaji M. R. Prantil-Baun R. Novak R. (2020). Quantitative prediction of human pharmacokinetic responses to drugs via fluidically coupled vascularized organ chips. Nat. Biomed. Eng. 4, 421–436. 10.1038/s41551-019-0498-9 31988459 PMC8011576

[B45] Holton A. B. Sinatra F. L. Kreahling J. Conway A. J. Landis D. A. Altiok S. (2017). Microfluidic biopsy trapping device for the real-time monitoring of tumor microenvironment. PLoS One 12, e0169797. 10.1371/journal.pone.0169797 28085924 PMC5235371

[B46] Huang J. L. Chen H. Z. Gao X. L. (2018). Lipid-coated calcium phosphate nanoparticle and beyond: a versatile platform for drug delivery. J. Drug Target 26, 398–406. 10.1080/1061186x.2017.1419360 29258343

[B47] Hughes C. S. Postovit L. M. Lajoie G. A. (2010). Matrigel: a complex protein mixture required for optimal growth of cell culture. Proteomics 10, 1886–1890. 10.1002/pmic.200900758 20162561

[B48] Huh D. Leslie D. C. Matthews B. D. Fraser J. P. Jurek S. Hamilton G. A. (2012). A human disease model of drug toxicity-induced pulmonary edema in a lung-on-a-chip microdevice. Sci. Transl. Med. 4, 159ra147. 10.1126/scitranslmed.3004249 PMC8265389 23136042

[B49] Huh D. Matthews B. D. Mammoto A. Montoya-Zavala M. Hsin H. Y. Ingber D. E. (2010). Reconstituting organ-level lung functions on a chip. Science 328, 1662–1668. 10.1126/science.1188302 20576885 PMC8335790

[B50] Humayun M. Chow C. W. Young E. W. K. (2018). Microfluidic lung airway-on-a-chip with arrayable suspended gels for studying epithelial and smooth muscle cell interactions. Lab. Chip 18, 1298–1309. 10.1039/c7lc01357d 29651473

[B51] Inamura K. Ishikawa Y. (2010). Lung cancer progression and metastasis from the prognostic point of view. Clin. Exp. Metastasis 27, 389–397. 10.1007/s10585-010-9313-4 20225084

[B52] Ingber D. E. (2020). Is it time for reviewer 3 to request human organ chip experiments instead of animal validation studies? Adv. Sci. (Weinh) 7, 2002030. 10.1002/advs.202002030 33240763 PMC7675190

[B53] Ingber D. E. (2022). Human organs-on-chips for disease modelling, drug development and personalized medicine. Nat. Rev. Genet. 23, 467–491. 10.1038/s41576-022-00466-9 35338360 PMC8951665

[B54] Jain A. Barrile R. van der Meer A. D. Mammoto A. Mammoto T. De Ceunynck K. (2018). Primary human lung alveolus-on-a-chip model of intravascular thrombosis for assessment of therapeutics. Clin. Pharmacol. Ther. 103, 332–340. 10.1002/cpt.742 28516446 PMC5693794

[B55] Jain A. Jain S. K. (2018). Advances in tumor targeted liposomes. Curr. Mol. Med. 18, 44–57. 10.2174/1566524018666180416101522 29663884

[B56] Jenkins R. W. Aref A. R. Lizotte P. H. Ivanova E. Stinson S. Zhou C. W. (2018). *Ex vivo* profiling of PD-1 blockade using organotypic tumor spheroids. Cancer Discov. 8, 196–215. 10.1158/2159-8290.cd-17-0833 29101162 PMC5809290

[B57] Jensen C. Teng Y. (2020). Is it time to start transitioning from 2D to 3D cell culture? Front. Mol. Biosci. 7, 33. 10.3389/fmolb.2020.00033 32211418 PMC7067892

[B58] Kapalczynska M. Kolenda T. Przybyla W. Zajaczkowska M. Teresiak A. Filas V. (2018). 2D and 3D cell cultures - a comparison of different types of cancer cell cultures. Arch. Med. Sci. 14, 910–919. 10.5114/aoms.2016.63743 30002710 PMC6040128

[B59] Katt M. E. Placone A. L. Wong A. D. Xu Z. S. Searson P. C. (2016). *In vitro* tumor models: advantages, disadvantages, variables, and selecting the right platform. Front. Bioeng. Biotechnol. 4, 12. 10.3389/fbioe.2016.00012 26904541 PMC4751256

[B60] Ke W. Yin W. Zha Z. Mukerabigwi J. F. Chen W. Wang Y. (2018). A robust strategy for preparation of sequential stimuli-responsive block copolymer prodrugs via thiolactone chemistry to overcome multiple anticancer drug delivery barriers. Biomaterials 154, 261–274. 10.1016/j.biomaterials.2017.11.006 29149720

[B61] Kilian K. A. Bugarija B. Lahn B. T. Mrksich M. (2010). Geometric cues for directing the differentiation of mesenchymal stem cells. Proc. Natl. Acad. Sci. U. S. A. 107, 4872–4877. 10.1073/pnas.0903269107 20194780 PMC2841932

[B62] Kim H. Sa J. K. Kim J. Cho H. J. Oh H. J. Choi D. H. (2022). Recapitulated crosstalk between cerebral metastatic lung cancer cells and brain perivascular tumor microenvironment in a microfluidic Co-culture chip. Adv. Sci. 9, e2201785. 10.1002/advs.202201785 PMC9353479 35657027

[B63] Kim S. Y. Moon A. (2012). Drug-induced nephrotoxicity and its biomarkers. Biomol. Ther. Seoul. 20, 268–272. 10.4062/biomolther.2012.20.3.268 24130922 PMC3794522

[B64] Ko J. Baldassano S. N. Loh P. L. Kording K. Litt B. Issadore D. (2018). Machine learning to detect signatures of disease in liquid biopsies - a user's guide. Lab. Chip 18, 395–405. 10.1039/c7lc00955k 29192299 PMC5955608

[B65] Kobayashi H. Turkbey B. Watanabe R. Choyke P. L. (2014). Cancer drug delivery: considerations in the rational design of nanosized bioconjugates. Bioconjug Chem. 25, 2093–2100. 10.1021/bc500481x 25385142 PMC4275162

[B66] Kobayashi Y. Mitsudomi T. (2016). Not all epidermal growth factor receptor mutations in lung cancer are created equal: perspectives for individualized treatment strategy. Cancer Sci. 107, 1179–1186. 10.1111/cas.12996 27323238 PMC5021039

[B67] Kohl Y. Biehl M. Spring S. Hesler M. Ogourtsov V. Todorovic M. (2021). Microfluidic *in vitro* platform for (Nano)Safety and (Nano)Drug efficiency screening. Small 17, e2006012. 10.1002/smll.202006012 33458959

[B68] Kourou K. Exarchos T. P. Exarchos K. P. Karamouzis M. V. Fotiadis D. I. (2015). Machine learning applications in cancer prognosis and prediction. Comput. Struct. Biotechnol. J. 13, 8–17. 10.1016/j.csbj.2014.11.005 25750696 PMC4348437

[B69] Kunimasa K. Goto T. (2020). Immunosurveillance and immunoediting of lung cancer: current perspectives and challenges. Int. J. Mol. Sci. 21, 597. 10.3390/ijms21020597 31963413 PMC7014343

[B70] Lai B. F. L. Lu R. X. Z. Hu Y. S. Huyer L. D. Dou W. Wang E. Y. (2020). Recapitulating pancreatic tumor microenvironment through synergistic use of patient organoids and organ-on-a-chip vasculature. Adv. Funct. Mater 30, 2000545. 10.1002/adfm.202000545 33692660 PMC7939064

[B71] Lai Benjamin F. L. Lu Rick X. Hu Y. Davenport H. L. Dou W. Wang E. Y. (2020). Recapitulating pancreatic tumor microenvironment through synergistic use of patient organoids and organ-on-a-chip vasculature. Adv. Funct. Mater 30, 2000545. 10.1002/adfm.202000545 33692660 PMC7939064

[B72] Le V. M. Lang M. D. Shi W. B. Liu J. W. (2016). A collagen-based multicellular tumor spheroid model for evaluation of the efficiency of nanoparticle drug delivery. Artif. Cells Nanomed Biotechnol. 44, 540–544. 10.3109/21691401.2014.968820 25315504

[B73] Li C. Wang J. Wang Y. Gao H. Wei G. Huang Y. (2019). Recent progress in drug delivery. Acta Pharm. Sin. B 9, 1145–1162. 10.1016/j.apsb.2019.08.003 31867161 PMC6900554

[B74] Liao W. Wang J. Xu J. You F. Pan M. Xu X. (2019). High-throughput three-dimensional spheroid tumor model using a novel stamp-like tool. J. Tissue Eng. 10, 204173141988918. 10.1177/2041731419889184 PMC6886283 31827757

[B75] Liguori G. R. Jeronimus B. F. de Aquinas Liguori T. T. Moreira L. F. P. Harmsen M. C. (2017). <sup/>Ethical issues in the use of animal models for tissue engineering: reflections on legal aspects, moral theory, three rs strategies, and harm–benefit analysis. Tissue Eng. Part C Methods 23, 850–862. 10.1089/ten.tec.2017.0189 28756735

[B76] Lim Z. F. Ma P. C. (2019). Emerging insights of tumor heterogeneity and drug resistance mechanisms in lung cancer targeted therapy. J. Hematol. Oncol. 12, 134. 10.1186/s13045-019-0818-2 31815659 PMC6902404

[B77] Liu J. Li D. Luo H. Zhu X. (2019). Circular RNAs: the star molecules in cancer. Mol. Asp. Med. 70, 141–152. 10.1016/j.mam.2019.10.006 31676107

[B78] Liu J. J. Li M. H. Luo Z. Dai L. L. Guo X. M. Cai K. Y. (2017). Design of nanocarriers based on complex biological barriers *in vivo* for tumor therapy. Nano Today 15, 56–90. 10.1016/j.nantod.2017.06.010

[B79] Liu J. J. Zhou B. Guo Y. L. Zhang A. M. Yang K. He Y. (2021). SR-A-Targeted nanoplatform for sequential photothermal/photodynamic ablation of activated macrophages to alleviate atherosclerosis. Acs Appl. Mater Inter 13, 29349–29362. 10.1021/acsami.1c06380 34133141

[B80] Low L. A. Tagle D. A. (2017). Tissue chips - innovative tools for drug development and disease modeling. Lab. Chip 17, 3026–3036. 10.1039/c7lc00462a 28795174 PMC5621042

[B81] Lynch T. J. Bell D. W. Sordella R. Gurubhagavatula S. Okimoto R. A. Brannigan B. W. (2004). Activating mutations in the epidermal growth factor receptor underlying responsiveness of non-small-cell lung cancer to gefitinib. New Engl. J. Med. 350, 2129–2139. 10.1056/nejmoa040938 15118073

[B82] Majidinia M. Mirza-Aghazadeh-Attari M. Rahimi M. Mihanfar A. Karimian A. Safa A. (2020). Overcoming multidrug resistance in cancer: recent progress in nanotechnology and new horizons. IUBMB Life 72, 855–871. 10.1002/iub.2215 31913572

[B83] Marcazzan S. Dadbin A. Brachi G. Blanco E. Varoni E. M. Lodi G. (2021). Development of lung metastases in mouse models of tongue squamous cell carcinoma. Oral Dis. 27, 494–505. 10.1111/odi.13592 32767730

[B84] Meador C. B. Hata A. N. (2020). Acquired resistance to targeted therapies in NSCLC: updates and evolving insights. Pharmacol. Ther. 210, 107522. 10.1016/j.pharmthera.2020.107522 32151666 PMC8675642

[B85] Metzger R. J. Klein O. D. Martin G. R. Krasnow M. A. (2008). The branching programme of mouse lung development. Nature 453, 745–750. 10.1038/nature07005 18463632 PMC2892995

[B86] Miller K. D. Nogueira L. Mariotto A. B. Rowland J. H. Yabroff K. R. Alfano C. M. (2019). Cancer treatment and survivorship statistics, 2019. CA Cancer J. Clin. 69, 363–385. 10.3322/caac.21565 31184787

[B87] Moosavian S. A. Sahebkar A. (2019). Aptamer-functionalized liposomes for targeted cancer therapy. Cancer Lett. 448, 144–154. 10.1016/j.canlet.2019.01.045 30763718

[B88] Nawroth J. C. Barrile R. Conegliano D. van Riet S. Hiemstra P. S. Villenave R. (2019). Stem cell-based Lung-on-Chips: the best of both worlds? Adv. Drug Deliv. Rev. 140, 12–32. 10.1016/j.addr.2018.07.005 30009883 PMC7172977

[B89] Nikolic M. Sustersic T. Filipovic N. (2018). *In vitro* models and on-chip systems: biomaterial interaction studies with tissues generated using lung epithelial and liver metabolic cell lines. Front. Bioeng. Biotechnol. 6, 120. 10.3389/fbioe.2018.00120 30234106 PMC6129577

[B90] Oliver C. R. Altemus M. A. Westerhof T. M. Cheriyan H. Cheng X. Dziubinski M. (2019). A platform for artificial intelligence based identification of the extravasation potential of cancer cells into the brain metastatic niche. Lab. Chip 19, 1162–1173. 10.1039/c8lc01387j 30810557 PMC6510031

[B91] Ozyurt C. Uludag I. Ince B. Sezginturk M. K. (2023). Lab-on-a-chip systems for cancer biomarker diagnosis. J. Pharm. Biomed. Anal. 226, 115266. 10.1016/j.jpba.2023.115266 36706542

[B92] Park J. Choi Y. Chang H. Um W. Ryu J. H. Kwon I. C. (2019). Alliance with EPR effect: combined strategies to improve the EPR effect in the tumor microenvironment. Theranostics 9, 8073–8090. 10.7150/thno.37198 31754382 PMC6857053

[B93] Park J. Y. Ryu H. Lee B. Ha D. H. Ahn M. Kim S. (2018). Development of a functional airway-on-a-chip by 3D cell printing. Biofabrication 11, 015002. 10.1088/1758-5090/aae545 30270851

[B94] Park S. Kim T. H. Kim S. H. You S. Jung Y. (2021). Three-dimensional vascularized lung cancer-on-a-chip with lung extracellular matrix hydrogels for *in vitro* screening. Cancers (Basel) 13, 3930. 10.3390/cancers13163930 34439103 PMC8393390

[B95] Parums D. V. (2022). Editorial: recent approval of sotorasib as the first targeted therapy for KRAS G12C-mutated advanced non-small cell lung cancer (NSCLC). Med. Sci. Monit. 28, e938746. 10.12659/msm.938746 36317327 PMC9636839

[B96] Qin S. Y. Cheng Y. J. Lei Q. Zhang A. Q. Zhang X. Z. (2018). Combinational strategy for high-performance cancer chemotherapy. Biomaterials 171, 178–197. 10.1016/j.biomaterials.2018.04.027 29698868

[B97] Ran R. Wang H. F. Hou F. Liu Y. Hui Y. Petrovsky N. (2019). A microfluidic tumor-on-a-chip for assessing multifunctional liposomes' tumor targeting and anticancer efficacy. Adv. Healthc. Mater 8, e1900015. 10.1002/adhm.201900015 30868753

[B98] Ren K. Zhou J. Wu H. (2013). Materials for microfluidic chip fabrication. Acc. Chem. Res. 46, 2396–2406. 10.1021/ar300314s 24245999

[B99] Sachs N. de Ligt J. Kopper O. Gogola E. Bounova G. Weeber F. (2018). A living biobank of breast cancer organoids captures disease heterogeneity. Cell 172, 373–386 e10. 10.1016/j.cell.2017.11.010 29224780

[B100] Sander C. Wallenborn M. Brandt V. P. Ahnert P. Reuschel V. Eisenloffel C. (2019). Central neurocytoma: SNP array analyses, subtel FISH, and review of the literature. Pathol. Res. Pract. 215, 152397. 10.1016/j.prp.2019.03.025 31000381

[B101] Sellgren K. L. Butala E. J. Gilmour B. P. Randell S. H. Grego S. (2014). A biomimetic multicellular model of the airways using primary human cells. Lab. Chip 14, 3349–3358. 10.1039/c4lc00552j 25000964

[B102] Sepesi B. Cascone T. Chun S. G. Altan M. Le X. (2020). Emerging therapies in thoracic malignancies-immunotherapy, targeted therapy, and T-cell therapy in non-small cell lung cancer. Surg. Oncol. Clin. N. Am. 29, 555–569. 10.1016/j.soc.2020.06.009 32883458 PMC7388816

[B103] Shanker M. Willcutts D. Roth J. A. Ramesh R. (2010). Drug resistance in lung cancer. Lung Cancer (Auckl) 1, 23–36. 10.2147/lctt.s6861 28210104 PMC5312467

[B104] Shi Y. van der Meel R. Chen X. Lammers T. (2020). The EPR effect and beyond: strategies to improve tumor targeting and cancer nanomedicine treatment efficacy. Theranostics 10, 7921–7924. 10.7150/thno.49577 32685029 PMC7359085

[B105] Shigeto H. Yamada E. Kitamatsu M. Ohtsuki T. Iizuka A. Akiyama Y. (2020). Analysis of single nucleotide-mutated single-cancer cells using the combined technologies of single-cell microarray chips and peptide nucleic acid-DNA probes. Micromachines (Basel) 11, 628. 10.3390/mi11070628 32605095 PMC7407912

[B106] Shirali A. C. Sprangers B. (2022). Cancer drug dosing in chronic kidney disease and dialysis. Adv. Chronic Kidney Dis. 29, 208–216 e1. 10.1053/j.ackd.2021.12.002 35817528

[B107] Shrestha J. Razavi Bazaz S. Aboulkheyr Es H. Yaghobian Azari D. Thierry B. Ebrahimi Warkiani M. (2020). Lung-on-a-chip: the future of respiratory disease models and pharmacological studies. Crit. Rev. Biotechnol. 40, 213–230. 10.1080/07388551.2019.1710458 31906727

[B108] Shrivastava P. Singh S. M. Singh N. (2004). Effect of thymosin alpha 1 on the antitumor activity of tumor-associated macrophage-derived dendritic cells. J. Biomed. Sci. 11, 623–630. 10.1007/bf02256128 15316138

[B109] Si L. Bai H. Rodas M. Cao W. Oh C. Y. Jiang A. (2021). A human-airway-on-a-chip for the rapid identification of candidate antiviral therapeutics and prophylactics. Nat. Biomed. Eng. 5, 815–829. 10.1038/s41551-021-00718-9 33941899 PMC8387338

[B110] Siegel R. L. Miller K. D. Fuchs H. E. Jemal A. (2022). Cancer statistics, 2022. CA Cancer J. Clin. 72, 7–33. 10.3322/caac.21708 35020204

[B111] Sindhwani S. Syed A. M. Ngai J. Kingston B. R. Maiorino L. Rothschild J. (2020). The entry of nanoparticles into solid tumours. Nat. Mater 19, 566–575. 10.1038/s41563-019-0566-2 31932672

[B112] Singh D. K. Ahrens C. C. Li W. Vanapalli S. A. (2017). Label-free, high-throughput holographic screening and enumeration of tumor cells in blood. Lab. Chip 17, 2920–2932. 10.1039/c7lc00149e 28718848

[B113] Song Y. Kim J. S. Kim S. H. Park Y. K. Yu E. Kim K. H. (2018). Patient-derived multicellular tumor spheroids towards optimized treatment for patients with hepatocellular carcinoma. J. Exp. Clin. Cancer Res. 37, 109. 10.1186/s13046-018-0752-0 29801504 PMC5970513

[B114] Sontheimer-Phelps A. Hassell B. A. Ingber D. E. (2019). Modelling cancer in microfluidic human organs-on-chips. Nat. Rev. Cancer 19, 65–81. 10.1038/s41568-018-0104-6 30647431

[B115] Stockwell B. R. (2022). Ferroptosis turns 10: emerging mechanisms, physiological functions, and therapeutic applications. Cell 185, 2401–2421. 10.1016/j.cell.2022.06.003 35803244 PMC9273022

[B116] Sun R. Hou Z. Zhang Y. Jiang B. (2022). Drug resistance mechanisms and progress in the treatment of EGFR‑mutated lung adenocarcinoma (Review). Oncol. Lett. 24, 408. 10.3892/ol.2022.13528 36245822 PMC9555020

[B117] Tan C. L. Chan Y. Candasamy M. Chellian J. Madheswaran T. Sakthivel L. P. (2022a). Unravelling the molecular mechanisms underlying chronic respiratory diseases for the development of novel therapeutics via *in vitro* experimental models. Eur. J. Pharmacol. 919, 174821. 10.1016/j.ejphar.2022.174821 35151643

[B118] Tan J. Sun X. Zhang J. Li H. Kuang J. Xu L. (2022b). Exploratory evaluation of EGFR-targeted anti-tumor drugs for lung cancer based on lung-on-a-chip. Biosens. (Basel) 12, 618. 10.3390/bios12080618 PMC9405841 36005014

[B119] Tian C. Zheng S. Liu X. Kamei K. I. (2022). Tumor-on-a-chip model for advancement of anti-cancer nano drug delivery system. J. Nanobiotechnology 20, 338. 10.1186/s12951-022-01552-0 35858898 PMC9301849

[B120] Tien J. C. Chugh S. Goodrum A. E. Cheng Y. Mannan R. Zhang Y. (2021). AGO2 promotes tumor progression in KRAS-driven mouse models of non-small cell lung cancer. Proc. Natl. Acad. Sci. U. S. A. 118, e2026104118. 10.1073/pnas.2026104118 33972443 PMC8157917

[B121] Trietsch S. J. Naumovska E. Kurek D. Setyawati M. C. Vormann M. K. Wilschut K. J. (2017). Membrane-free culture and real-time barrier integrity assessment of perfused intestinal epithelium tubes. Nat. Commun. 8, 262. 10.1038/s41467-017-00259-3 28811479 PMC5557798

[B122] Van Zundert I. Fortuni B. Rocha S. (2020). From 2D to 3D cancer cell models-the enigmas of drug delivery research. Nanomater. (Basel) 10, 2236. 10.3390/nano10112236 PMC7696259 33187231

[B123] Veith I. Nurmik M. Mencattini A. Damei I. Lansche C. Brosseau S. (2024). Assessing personalized responses to anti-PD-1 treatment using patient-derived lung tumor-on-chip. Cell Rep. Med., 101549. 10.1016/j.xcrm.2024.101549 38703767 PMC11148770

[B124] Vlachogiannis G. Hedayat S. Vatsiou A. Jamin Y. Fernandez-Mateos J. Khan K. (2018). Patient-derived organoids model treatment response of metastatic gastrointestinal cancers. Science 359, 920–926. 10.1126/science.aao2774 29472484 PMC6112415

[B125] Vo Q. Carlson K. A. Chiknas P. M. Brocker C. N. DaSilva L. Clark E. (2024). On-chip reconstitution of uniformly shear-sensing 3D matrix-embedded multicellular blood microvessel. Adv. Funct. Mater 34, 2304630. 10.1002/adfm.202304630 38465199 PMC10923530

[B126] Walsh C. L. Babin B. M. Kasinskas R. W. Foster J. A. McGarry M. J. Forbes N. S. (2009). A multipurpose microfluidic device designed to mimic microenvironment gradients and develop targeted cancer therapeutics. Lab. Chip 9, 545–554. 10.1039/b810571e 19190790 PMC2855303

[B127] Wang D. Li X. Jiao D. Cai Y. Qian L. Shen Y. (2023b). LCN2 secreted by tissue-infiltrating neutrophils induces the ferroptosis and wasting of adipose and muscle tissues in lung cancer cachexia. J. Hematol. Oncol. 16, 30. 10.1186/s13045-023-01429-1 36973755 PMC10044814

[B128] Wang G. Xie L. Li B. Sang W. Yan J. Li J. (2021). A nanounit strategy reverses immune suppression of exosomal PD-L1 and is associated with enhanced ferroptosis. Nat. Commun. 12, 5733. 10.1038/s41467-021-25990-w 34593794 PMC8484261

[B129] Wang N. Ma T. Yu B. (2023a). Targeting epigenetic regulators to overcome drug resistance in cancers. Signal Transduct. Target Ther. 8, 69. 10.1038/s41392-023-01341-7 36797239 PMC9935618

[B130] Wang Q. Wang Y. Ding J. Wang C. Zhou X. Gao W. (2020). A bioorthogonal system reveals antitumour immune function of pyroptosis. Nature 579, 421–426. 10.1038/s41586-020-2079-1 32188939

[B131] Wlodkowic D. Cooper J. M. (2010). Tumors on chips: oncology meets microfluidics. Curr. Opin. Chem. Biol. 14, 556–567. 10.1016/j.cbpa.2010.08.016 20832352

[B132] Wu P. Gao W. Su M. Nice E. C. Zhang W. Lin J. (2021). Adaptive mechanisms of tumor therapy resistance driven by tumor microenvironment. Front. Cell Dev. Biol. 9, 641469. 10.3389/fcell.2021.641469 33732706 PMC7957022

[B133] Xu Z. Gao Y. Hao Y. Li E. Wang Y. Zhang J. (2013). Application of a microfluidic chip-based 3D co-culture to test drug sensitivity for individualized treatment of lung cancer. Biomaterials 34, 4109–4117. 10.1016/j.biomaterials.2013.02.045 23473962

[B134] Xu Z. Li E. Guo Z. Yu R. Hao H. Xu Y. (2016). Design and construction of a multi-organ microfluidic chip mimicking the *in vivo* microenvironment of lung cancer metastasis. ACS Appl. Mater Interfaces 8, 25840–25847. 10.1021/acsami.6b08746 27606718

[B135] Yang S. Zhang T. Ge Y. Cheng Y. Yin L. Pu Y. (2023). Sentinel supervised lung-on-a-chip: a new environmental toxicology platform for nanoplastic-induced lung injury. J. Hazard Mater 458, 131962. 10.1016/j.jhazmat.2023.131962 37406524

[B136] Yang X. Li K. Zhang X. Liu C. Guo B. Wen W. (2018). Nanofiber membrane supported lung-on-a-chip microdevice for anti-cancer drug testing. Lab. Chip 18, 486–495. 10.1039/c7lc01224a 29309077

[B137] Yang Y. Yu C. (2016). Advances in silica based nanoparticles for targeted cancer therapy. Nanomedicine 12, 317–332. 10.1016/j.nano.2015.10.018 26706409

[B138] Ye Z. Huang Y. Ke J. Zhu X. Leng S. Luo H. (2021). Breakthrough in targeted therapy for non-small cell lung cancer. Biomed. Pharmacother. 133, 111079. 10.1016/j.biopha.2020.111079 33378976

[B139] Yun C. H. Mengwasser K. E. Toms A. V. Woo M. S. Greulich H. Wong K. K. (2008). The T790M mutation in EGFR kinase causes drug resistance by increasing the affinity for ATP. P Natl. Acad. Sci. U. S. A. 105, 2070–2075. 10.1073/pnas.0709662105 PMC2538882 18227510

[B140] Zamprogno P. Wuthrich S. Achenbach S. Thoma G. Stucki J. D. Hobi N. (2021). Second-generation lung-on-a-chip with an array of stretchable alveoli made with a biological membrane. Commun. Biol. 4, 168. 10.1038/s42003-021-01695-0 33547387 PMC7864995

[B141] Zhang J. Huang D. Saw P. E. Song E. (2022). Turning cold tumors hot: from molecular mechanisms to clinical applications. Trends Immunol. 43, 523–545. 10.1016/j.it.2022.04.010 35624021

[B142] Zhang Y. S. Aleman J. Shin S. R. Kilic T. Kim D. Mousavi Shaegh S. A. (2017). Multisensor-integrated organs-on-chips platform for automated and continual *in situ* monitoring of organoid behaviors. Proc. Natl. Acad. Sci. U. S. A. 114, E2293–E2302. 10.1073/pnas.1612906114 28265064 PMC5373350

[B143] Zhang Z. Wang H. Ding Q. Xing Y. Xu Z. Lu C. (2018). Establishment of patient-derived tumor spheroids for non-small cell lung cancer. PLoS One 13, e0194016. 10.1371/journal.pone.0194016 29543851 PMC5854348

[B144] Zhao L. Cao Y. J. (2019). Engineered T cell therapy for cancer in the clinic. Front. Immunol. 10, 2250. 10.3389/fimmu.2019.02250 31681259 PMC6798078

[B145] Zheng J. F. He S. Zeng Z. Gu X. Cai L. Qi G. (2019). PMPCB silencing sensitizes HCC tumor cells to sorafenib therapy. Mol. Ther. 27, 1784–1795. 10.1016/j.ymthe.2019.06.014 31337603 PMC6822227

[B146] Zhou H. Fu H. Liu H. Shao X. Cai W. (2022). Uncovering the mechanism of drug resistance caused by the T790M mutation in EGFR kinase from absolute binding free energy calculations. Front. Mol. Biosci. 9, 922839. 10.3389/fmolb.2022.922839 35707225 PMC9189374

